# Post-Translational Modifications of Extracellular Proteasome

**DOI:** 10.3390/molecules25153504

**Published:** 2020-07-31

**Authors:** Anna S. Tsimokha, Tatiana O. Artamonova, Egor E. Diakonov, Mikhail A. Khodorkovskii, Alexey N. Tomilin

**Affiliations:** 1 Institute of Cytology of the Russian Academy of Sciences, 4 Tikhoretsky Ave., 194064 Saint-Petersburg, Russia; artamonova@nanobio.spbstu.ru (T.O.A.); e.diakonov@incras.ru (E.E.D.); nanobio@nanobio.spbstu.ru (M.A.K.); a.tomilin@incras.ru (A.N.T.); 2 Institute of Nanobiotechnologies, Peter the Great St. Petersburg Polytechnic University, 29 Polytechnicheskaya Str., 195251 Saint-Petersburg, Russia

**Keywords:** affinity purification, extracellular proteasome, fourier transform ion cyclotron mass spectrometry (FT-ICR MS), human leukemia K562 cells, matrix-assisted laser desorption/ionization (MALDI), post-translational modifications (PTMs)

## Abstract

The ubiquitin-proteasome system (UPS) is one of the major protein degradation pathways in eukaryotic cells. Abnormal functioning of this system has been observed in cancer and neurological diseases. The 20S proteasomes, essential components of the UPS, are present not only within the cells but also in the extracellular space, and their concentration in blood plasma has been found to be elevated and dependent upon the disease state, being of prognostic significance in patients suffering from cancer, liver diseases, and autoimmune diseases. However, functions of extracellular proteasomes and mechanisms of their release by cells remain largely unknown. The main mechanism of proteasome activity regulation is provided by modulation of their composition and post-translational modifications (PTMs). Moreover, diverse PTMs of proteins are known to participate in the loading of specific elements into extracellular vesicles. Since previous studies have revealed that the transport of extracellular proteasomes may occur via extracellular vesicles, we have set out to explore the PTMs of extracellular proteasomes in comparison to cellular counterparts. In this work, cellular and extracellular proteasomes were affinity purified and separated by SDS-PAGE for subsequent trypsinization and matrix-assisted laser desorption/ionization (MALDI) Fourier transform ion cyclotron resonance (FT-ICR) mass spectrometry (MS) analysis. In total, we could identify 64 and 55 PTM sites in extracellular and cellular proteasomes, respectively, including phosphorylation, ubiquitination, acetylation, and succinylation. We observed novel sites of acetylation at K238 and K192 of the proteasome subunits β2 and β3, respectively, that are specific for extracellular proteasomes. Moreover, cellular proteasomes show specific acetylation at K227 of α2 and ubiquitination at K201 of β3. Interestingly, succinylation of β6 at the residue K228 seems not to be present exclusively in extracellular proteasomes, whereas both extracellular and cellular proteasomes may also be acetylated at this site. The same situation takes place at K201 of the β3 subunit where ubiquitination is seemingly specific for cellular proteasomes. Moreover, crosstalk between acetylation, ubiquitination, and succinylation has been observed in the subunit α3 of both proteasome populations. These data will serve as a basis for further studies, aimed at dissection of the roles of extracellular proteasome-specific PTMs in terms of the function of these proteasomes and mechanism of their transport into extracellular space.

## 1. Introduction

The proteasome is a multisubunit protein complex that degrades proteins in eukaryotic cells in a ubiquitin-dependent manner. The precisely regulated ubiquitin-dependent proteolysis maintains normal cell cycle and survival. The term “proteasome” refers to both 26S proteasome and 20S proteasome. The 26S proteasome is an ATP-dependent 2.5-MDa proteolytic complex that consists of the 20S core particle and different regulatory particles, including the 19S regulatory particles located at one or both sides of the core particle [1]. The 19S regulator may include at least 19 variable subunits and has a molecular mass of 900 kDa [2]. The 19S particle recognizes and unwinds polyubiquitinated substrates, detaches ubiquitin monomers, and regulates substrate entry into 20S proteasome [3]. The cylinder-shaped 20S proteasome complex consists of seven duplicated α- and β-subunits with molecular masses of 20–35 kDa, stacked into four hetero-heptameric rings [4]. There are only three catalytically active β-subunits in higher eukaryotes 20S proteasome: β1, β2, β5 with caspase-, trypsin-, and chymotrypsin-like activities, respectively. The 20S proteasome degrades the substrate, generating short peptides with average size of 8–12 amino acids. These peptides are subsequently degraded into amino acids by aminopeptidases or presented on the cell surface by MHC I.

The proteasomes are also present in extracellular space: blood plasma [5], cerebrospinal [6] and alveolar fluids [7], as well as conditioned media from human cancer cell lines [8,9,10]. The extracellular proteasomes are represented solely by proteolitically active 20S core particles, as it has been revealed by electron microscopy [11] and mass-spectrometry [9,10]. A correlation between the concentration of extracellular proteasomes in blood plasma and oncological disease severity was shown [5,12,13,14]. A similar correlation was also shown in patients with liver disorders [5] and some autoimmune diseases [15]. It is important to note that the extracellular proteasome concentration can be considered as a prognostic parameter in some types of cancer [13,16,17].

Although extracellular proteasomes have been initially found in human blood plasma [5], the functions of this proteasome class in extracellular space still remain unknown. The origin of the extracellular proteasomes is also obscure and since proteasomes cannot be secreted by ER-to-Golgi pathway [18], a passive mechanism of proteasome release from disintegrating tumor cells has been proposed [5]. On the contrary, there was some evidence of an active release of proteasomes via extracellular vesicles (microparticles or exosomes) [6,19,20,21,22].

The main mechanism of regulation of proteasome activity, as it is presently known, is mediated by modulations of composition [23], gene regulation [24], associations with regulatory proteins [25] and post-translational modifications (PTMs) of proteasome subunits [26,27]. PTMs are also known to control proteasome activity, stability [28,29], assembly [30], and subcellular localization [31,32,33,34]. On the other hand, diverse PTMs of proteins participate in the loading of specific elements onto extracellular vesicles [35]. Therefore, to understand the functions of extracellular proteasomes and the mechanism of their release, it is highly relevant to identify specific PTMs.

In the current study, we have compared PTMs of cellular and extracellular proteasome complex affinity, purified from human K562 cells and medium, conditioned by these cells. We report potential extracellular proteasome-specific PTMs and discuss their possible impact on extracellular proteasome functions and the mechanism of their release.

## 2. Results and Discussion

**Figure 1:**
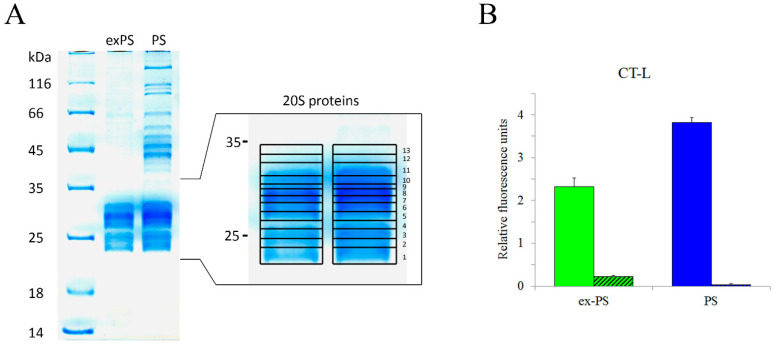
Affinity-purified extracellular and cellular proteasomes from conditioned medium (CM) and β7-HTBH K562 cells preserve chymotrypsin-like peptidase activity. (**A**) Proteins from affinity-purified cellular (PS) and extracellular (ex-PS) proteasomes (10 μg) were separated by SDS-PAGE and visualized with Coomassie Blue. Positions of 19S and 20S subcomplexes in the gel are shown. 20S proteasome proteins were cut into 13 pieces, which were then in-gel digested with trypsin. The peptide mixture was analyzed by matrix-assisted laser desorption/ionization Fourier transform ion cyclotron resonance mass spectrometry (MALDI FT-ICR MS). (**B**) Comparison of the purified intra- and extracellular proteasomes (1 μg) for chymotrypsin-like (CT-L) activity in the presence or absence of proteasome inhibitor MG132, determined by fluorometric quantification of the substrate Suc-LLVY-AMC (N-Succinyl-Leu-Leu-Val-Tyr-7-amino-4-methylcoumarin), using 380 nm excitation/440 nm emission, respectively. The results are presented in the Y-axis as relative fluorescence units. The control is Suc-LLVY-AMC background fluorescence.

We have previously demonstrated, using mass spectrometry, a number of new sites of phosphorylation, ubiquitination, and N-terminal modification for several subunits of cellular proteasome [36,37]. To define potential PTMs specific for extracellular proteasomes, we sought to compare the PTMs of cellular and extracellular proteasomes. To isolate proteasome complexes, we used previously generated human leukemia cell line K562-β7-HTBH stably expressing β-type subunit β7 (*PSMB4*) of the inner rings of the 20S proteolytic proteasome core tagged at its C-terminus, with the HTBH polypeptide consisting of two hexahistidine tags (H), TEV cleavage site (T), and a bacterially derived peptide that induces biotinylation in vivo (B). The tag allows two-step affinity purification of proteasomes from mammalian cells via (1) high-affinity binding of streptavidin–agarose beads to biotinylated regions of the HTBH tag followed by (2) its cleavage from the β7 subunit by TEV-protease. Since proteins from serum-containing culture media often contaminate samples of purified extracellular proteasomes, serum-free medium supplied with insulin, transferrin, and selenium has been applied. To exclude the possibility that any kind of cell death contributes to the serum starvation-induced release of extracellular proteasomes by cells, we have measured the amount of living and dead cells using Muse Count and Viability Assay. There were no significant effects of serum starvation on cell viability. The purified samples of proteasomes have been analyzed by SDS-PAGE, thereby confirming their intact state (Figure 1A). A lack of 19S regulatory particle proteins in samples of extracellular proteasomes has been observed (Figure 1A), consistent with our previous results [9,10].

In order to evaluate functionality of the affinity-purified cellular and extracellular proteasomes, we assessed their CT-like activity, using a fluorogenic peptidase assay (Figure 1B). The fluorescence measured in the presence of the proteasome inhibitor MG132, which was used as a control for the specificity of proteasomal activity.

The separation of cellular and extracellular proteasomes (Figure 1A) for subsequent trypsinization and MALDI-FT-ICR-MS-analysis was achieved via an SDS-PAGE. Mass-spectra were then searched against protein sequences from Swiss–Prot database, using Mascot and Protein Prospector MS-Fit software. All 14 subunits of 20S-proteasome—seven α and seven β subunits—have been observed.

Three biological replicates were collected for each proteasome sample, and the overlapping peptides, detected across 2 out of the 3 biological replicates, were used for the analysis of PTMs (Supplementary Materials Tables S1 and S2). In total, we could identify 64 and 55 PTM sites for extracellular and cellular proteasomes, respectively, including phosphorylation, ubiquitination, acetylation, and succinylation (Table 1 and Supplementary Materials Table S3). In some cases, however, it was not possible to pinpoint modification sites within given peptides due to the nature of MS-analysis, which is different from the MS/MS-analysis with sequencing capability (Supplementary Materials Table S2). Many of the identified PTMs have been previously reported in human proteasome [25,38].

According to our data, 26 out of 64 identified PTMs seem to be present only in extracellular proteasomes (Supplementary Materials Table S3). To further determine PTMs specific for extracellular proteasome, we compared our data with PTMs listed in PhosphoSitePlus (phosphosite.org) and recently published ones [25,38]. Since all of these PTMs have been previously detected in cellular proteasomes, they cannot be considered specific for extracellular ones. Therefore, we excluded from the list of candidates the S201-p of α4, S79-p of α5, S40-p and T42-p of α6, and S23-p and K198-ub of β4. It is important to note that several PTMs (K205-ac of α3, K239-ac and S198-p of α5, K192-ac of α7, T121-p, T233-p and K237-ac of β2, S181-p of β3, and K29-ac of β4) have only been previously detected in mouse or rat proteasomes. For example, despite the fact that the phosphorylation site S181 of human proteasome subunit β3 was not previously reported in human, it was identified in mouse proteasomes (Supplementary Materials Table S3). Likewise, the subunit β2 was shown to be phosphorylated at T233 in mouse and acetylated at K237 in rat and mouse, while ubiquitination of β2 at this lysin residue was determined in human and rat. Moreover, acetylation at K239 of α5 and at K29 of β4 were also found in mouse and rat, respectively. It is important to stress, however, that most of the known PTMs on human proteasome are conserved in mouse and rat proteasome subunits. Therefore, we also excluded from the list of 26 extracellular proteasome PTMs those PTMs that were identified in mice or rat proteasomes, but not in human ones (K239-ac of α5, T233-p and K237-ac of β2, S181-p of β3, and K29-ac of β4). This allowed us to pinpoint 15 PTMs that could be specific for extracellular proteasome (Supplementary Materials Table S3).

**Figure 2:**
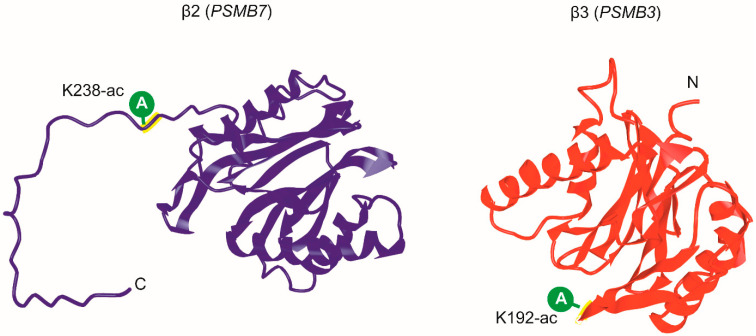
The structure of proteasome subunits with post-translational modifications detected by MALDI-ICR mass spectrometry. A—acetylation.

We considered only those PTMs that were found in two or three biological replicates, because we consider those statistically significant (Supplementary Materials Tables S1 and S2). However, the PTMs found in one biological replicate were also taken into account for extracellular proteasome. Next, we additionally excluded from consideration those PTMs of extracellular proteasomes that were identified only once during the analysis of intracellular counterparts in either technical or biological replicates (Supplementary Materials Table S1). By applying this principle, phosphorylation at Y228 of α4, acetylation and succinylation at K41 and K50, and succinylation at K62 of α6, succinylation at K208 of α7, phosphorylation at T61, T64, Y232 and acetylation at K225 of β2, ubiquitination at K130 of β5, phosphorylation at Y103 of β6, initially revealed in extracellular proteasomes (Table 1), were subsequently excluded from the list of extracellular proteasomes-specific candidate PTMs. Eventually, only two PTMs were identified as specific for extracellular proteasomes: acetylation at K238 of β2 and at K192 of β3 (Table 1 and Figure 2). In addition, according to our data, acetylation at K227 of α2 and ubiquitination at K201 of β3 are PTMs specific for intracellular proteasomes (Table 1).

Interestingly, succinylation of β6 at K228 residue seems not to be present only in extracellular proteasomes, whereas both extracellular and cellular proteasomes may also be acetylated at this site (Table 1). Subunit β5 of extracellular proteasome may be ubiquitinated at K130, and both extracellular and cellular proteasomes may also be acetylated at this site. Similarly, both proteasome populations revealed the ubiquitination of α2 subunit (K198); however, acetylation of this subunit at this site was identified only in cellular proteasomes. The crosstalk between acetylation and succinylation has been observed in the subunit α6 (K41 and 50) of extracellular proteasome populations. Moreover, the crosstalk between acetylation, ubiquitination, and succinylation has been observed in the subunit α3 (K222) of both proteasome populations.

It should be pointed out that a significant overlap between the ubiquitination and acetylation sites has been observed in human cardiac 20S proteasomes [39]. This crosstalk between acetylation, ubiquitination, and succinylation of proteasomal proteins may permit potential crosstalk in the regulation of proteasome functions.

Phosphorylation is a PTM by which a phospho-group binds to serine, threonine, and/or tyrosine residues of proteins; hence, many phosphoproteins are formed. Phosphorylation and its counterpart dephosphorylation are catalyzed by kinases and phosphatases, respectively, regulating protein function. Protein phosphorylation plays an important role in inter- and intracellular signal transduction. Proteins can acquire two negative charges by phosphorylation and form more than three hydrogen bonds. In this manner, proteins may change their modes of interaction with other proteins or ligands by phosphorylation. Almost all subunits of the yeast and human 26S proteasome have been shown to be phosphorylated [38,40]. Phosphorylation is one of the most frequent and best studied PTMs of the proteasome. The phosphorylation is involved in proteasome protease activity [40,41,42,43,44,45,46], stability [29,33], assembly [30], and subcellular localization [33]. More importantly, the proteasome is dynamically phosphorylated in a variety of physiological and pathological processes, including cell cycle, differentiation, metabolic changes, DNA damage and stress responses, and oncogenesis [38]. The functional roles of proteasome phosphorylation in these processes are, however, largely uncharacterized. For example, only a small part of the 455 identified phosphosites of the human proteasome has known functions [38]. In other words, there are many phosphorylation sites in each proteasome subunit, but the question arises as to whether all phosphorylation sites are functional. Given that proteins are analyzed by the protein shotgun method after digestion, it is often difficult to precisely determine the PTM status of proteins. Phosphorylation status may vary within the protein population, and during the shotgun analysis, this information might be lost. At the same time, 97% of known phosphosites on human 26S proteasome are conserved in mouse and rat proteasome subunits, and about 85% of them are found in zebrafish. However, the degree of site conservation drops considerably, to less than 50%, in yeast proteasomes.

Most studies suggest that, in general, proteasome phosphorylation and dephosphorylation are associated with increased and decreased proteasome activity, respectively [27]. Though in some cases, phosphorylation of a subunit led to the inhibition of the ChT-L activity of proteasomes in human and mouse cells [47,48]. However, it is not clear how different subunits contribute to the altered proteasome activity. Modifications may directly affect the proteolytic function of the proteasome as well as proteasome stability.

Ubiquitination occurs by the covalent bonding of the C-terminal glycine of an ubiquitin molecule to a lysine residue on a substrate protein. Ubiquitin, a protein comprising 76 amino acid residues, is covalently attached to lysine residues of target proteins by the ubiquitination machinery composed of ubiquitin-activating enzyme, ubiquitin-conjugating enzyme, and ubiquitin ligase. Ubiquitination is primarily involved in the selective degradation of proteins via the ubiquitin–proteasome system. Thus, polyubiquitinated proteins are recognized, deubiquitinated, and degraded by the proteasome. Failure of protein ubiquitination results in defects in biological processes such as protein quality control, cell cycle, apoptosis, DNA repair, signal transduction, and antigen presentation.

Although polyubiquitination is putatively involved in the selective degradation of proteins by the proteasome, ubiquitination can also affect proteins in other ways, such as altering activity, protein interactions, and cellular localization [49]. The 20S proteasome can also be regulated by ubiquitination [26].

Ubiquitination of the yeast 26S proteasome ubiquitin-receptor subunit Rpn10 inhibits the interaction of the 26S proteasome and polyubiquitinated substrates due to Rpn10 dissociation from the 26S proteasome [50]. Similarly, ubiquitination of Rpn13, a mammalian 26S proteasome ubiquitin-receptor subunit, strongly decreases the proteasome’s ability to bind and degrade ubiquitin-conjugated proteins, but does not affect its activity towards peptide substrates [51]. Furthermore, ubiquitination of the α2-subunit of the 20S proteasome leads to its interaction with a proteasome-associated protein ALAD, which specifically inhibits CT-like proteasome activity [52]. Moreover, ubiquitination of proteasomes in vitro reduces their CT-like activity [37]. Interestingly, Zong et al. suggested that the function of the proteasomes is regulated via crosstalk between acetylation and ubiquitination, because more than half of human cardiac 20S proteasome acetylation sites were also ubiquitinated [39]. Notably, lysine is the most frequently post-translationally modified residue of all proteinogenic amino acids, being a target for ubiquitination, acetylation, succinylation, and methylation.

Succinylation is a PTM by the addition of a succinyl group to lysine residue of proteins. Succinylation has been found in 13 of 35 subunits of the yeast 26S proteasome [53], but its biological significance is yet to be explored. Interestingly, there were overlapping succinylation and acetylation observed at the lysine residues of α3, α4, β3, β6, Rpt3, Rpn2, Rpn3, Rpn9, Rpn12, and Rpn13 [53]. The authors suggested a crosstalk between succinylation and acetylation as well as a relationship between phosphorylation and acetylation or ubiquitination and acetylation.

Even though succinylation occurs at low levels and is difficult to be detected by mass spectrometry, we detected seven novel succinylation sites at the α3, α4, α6, α7, and β6 subunits. Interestingly, succinylation of β6 at K228 residue seems to be absent from extracellular proteasomes (contrary to cellular ones), whereas both extracellular and cellular proteasomes may be acetylated at this site. Moreover, a crosstalk between acetylation, ubiquitination, and succinylation has been observed for the α3 subunit of both proteasome populations. The same situation takes place at the α6 subunit, where both lysin residue K41 and 50 are acetylated and succinylated in extracellular proteasomes.

Acetylation of proteasomes refers to the substitution of an acetyl group for an active hydrogen atom on the lysine residues of proteasomal subunits. In yeast, N^α^-acetyltransferase NAT1 deletion mutants lacking α3 N^α^-acetylation showed a higher chymotrypsin-like activity of the 20S proteasome than normal strains [54]. It was proposed that this effect might be caused by a change in the structure of the 20S proteasome, resulting in the opening of the channel into the core particle. Besides, it was shown that the deletion of the α3 N-terminal tail resulted in the opening of the 20S proteasome gate [55]. These observations might indicate that N^α^-acetylation of α3 regulates the 20S proteasome gate opening. However, this modification does not alter the activity of the 26S proteasome, because the 19S particle keeps the 20S proteasome gate open [56]. In mouse and human cells, acetylation of several proteasome subunits correlates with an increase in the proteasome activity [57]. In our study, extracellular proteasomes might have specific acetylation at K238 of β2 and at K192 of β3, which may also enhance or repress their activity.

Unfortunately, for the majority of PTMs, neither biological effect nor impact on proteasome functionality are presently known [24]. However, an elegant study reporting a compilation of 200,000 PTMs among 11 eukaryotic species predicted that only a small fraction of PTMs may (a) cross-regulate each other, (b) regulate domain activity, and (c) mediate protein–protein interactions [58]. According to the same study, PTMs with predicted functional significance are likely to be evolutionarily conserved and accumulate within regulatory regions or “hot spots” [58]. Indeed, it was previously shown that phosphorylation sites with known functions tended to be more conserved [59,60]. Because extracellular proteasomes were studied almost exclusively in human models, MS studies aimed at the identification of extracellular proteasome PTMs in other species would be highly beneficial.

Hence, we report, for the first time, PTMs specific for the population of extracellular proteasomes, which are the acetylation at K238 and K192 of the proteasome subunits β2 and β3, respectively. Moreover, we observed novel PTMs in cellular proteasomes, which are acetylation at K227 of α2 and ubiquitination at K201 of β3. The obtained data may be useful in future studies aiming at the dissection of molecular mechanisms of extracellular proteasome function and transport into extracellular space.

## 3. Material and Methods

### 3.1. Cell Culture Conditions and Cell Viability Assays

The human leukemia cell line K562 (Russian Cell Culture Collection, Institute of Cytology, Russia) was cultured in RPMI 1640 medium with 10% fetal bovine serum, 2 mM L-glutamine and 50 U/mL penicillin-streptomycin. This cell line was previously modified [36] to stably express genetic construction coding human proteasome subunit β7 (PSMB4).

To determine the number of viable cells present in a cell suspension, Muse Count and Viability kit (Muse Cell Analyzer, Merck, Darmstadt, Germany) were used.

### 3.2. Affinity Purification of Proteasome

Cellular proteasomes were purified from the whole-cell extract as described previously [61]. Briefly, K562-β7-HTBH cells were washed with cold PBS-buffer and then lysed for 30 min at 4 °C in the buffer A (50 mM Na-phosphate, pH 7.5, 100 mM NaCl, 10% glycerol, 5 mM ATP, 1 mM DTT, 5 mM MgCl_2_, 1x protease inhibitor cocktail (Roche), and 0.5% NP-40). After removal of cell debris (15,000 g, 30 min), the whole-cell extract was incubated with streptavidin–agarose beads (#20359, Thermo Scientific, Waltham, MA, USA) overnight at 4 °C. Proteasomes in complex with streptavidin agarose were precipitated by centrifugation and washed sequentially with 20 volumes of buffer A and 10 volumes of TEB buffer (50 mM Tris-HCl, pH 7.5, 10% glycerol). The 26S proteasome was eluted from the beads with a 0.1% tobacco etch virus (TEV)-protease (Sigma-Aldrich, St. Louis, MO, USA) in TEB buffer and concentrated using Amicon Ultra-0.5 centrifuge filters (100K NMWL, Merck, Darmstadt, Germany).

Extracellular proteasomes were purified from cell-conditioned medium as described previously [9]. Briefly, one day before cell-conditioned medium collection, the medium from the K562-β7-HTBH cells was removed; cells were washed twice with PBS and cultured overnight in serum-free medium RPMI 1640 supplemented with Insulin–Transferrin–Selenium (0.5 × 10^6^ cells in 1 mL of medium). On the day of collection, cell-conditioned medium was collected, pre-cleared (300 g, 10 min), centrifuged at 2000× *g* (20 min) to remove cell debris, then concentrated (100x) using Amicon Ultra-15 filters (100K NMWL, Merck). Subsequently, concentrated samples of cell-conditioned medium were incubated in the buffer A for 30 min at 4 °C, followed by disruption of extracellular vesicles by repeated freeze-thaw cycles, and then was incubated with streptavidin–agarose resin overnight at 4 °C. The beads were then washed twice with 20 bed volumes of the buffer I, followed by a final wash with 10 bed volumes of TEB buffer. To elute purified proteasomes, the streptavidin beads were incubated in 2 bed volumes of TEB buffer containing 1% TEV protease at 30 °C for 1.5 h. The eluted proteasomes were concentrated using Amicon Ultra-0.5 filters (100K NMWL, Merck).

The concentration of purified proteasomes was estimated using the Bradford assay.

### 3.3. Assay of Proteasome Proteolytic Activity

Chymotrypsin-like (CT-like) peptidase activity of the proteasomes was determined using Suc-LLVY-AMC (*N*-Succinyl-Leu-Leu-Val-Tyr-7-amino-4-methylcoumarin) substrate (Enzo Life Sciences, Lörrach, Germany) at a concentration of 0.25 mM in 50 mM Tris-HCl, pH 7.5, containing 5 mM MgCl2, 40 mM KCl, 1 mM DTT, 1 mM ATP at 37 °C for 45 min as described previously [62,63]. The reaction was stopped by adding an equal volume of stop solution (0.1 M sodium chloroacetate, 30 mM sodium acetate, 25 mM acetic acid, pH 5.0). Proteasome activity was monitored by measuring free AMC fluorescence, following subtraction of background fluorescence, using a FLUOstar Omega fluorometer (BMG Labtech, East Sussex, UK) with an excitation wavelength of 355 nm and an emission wavelength of 460 nm. The amount of liberated AMC was determined as fluorescence intensity. For a specificity control, the purified proteasomes were treated with 1 μM proteasome inhibitor MG132 or vehicle (DMSO).

### 3.4. MALDI FT-ICR Mass Spectrometry

A total of 10 μg of purified proteasomes was resolved on 13% SDS-PAGE and visualized by Coomassie staining. The protein-containing gel lane was then cut into pieces and incubated twice with 60 mM NH_4_HCO_3_ in 40% acetonitrile (ACN) for 20 min at 37 °C in a shaker for destaining. After drying the gel pieces with 100% ACN and vacuum evaporation, they were rehydrated in 50 mM NH_4_HCO_3_, 10% ACN containing 15 μg/mL proteomics-grade trypsin (Sigma Aldrich, St. Louis, MO, USA) and then incubated for 30 min on ice and 4 h at 37 °C. Extraction buffer (5% formic acid/ACN, 1:2 *v*/*v*) was added to each tube and incubated for 15 min at 37 °C in a shaker. Supernatant was collected, dried down in a vacuum centrifuge and dissolved in 0.1% TFA.

High-resolution mass spectra were recorded on a Fourier Transform Ion Cyclotron Resonance Mass Spectrometer (Varian 902-MS, Agilent Technologies, Santa Clara, CA, USA) equipped with a 9.4 T magnet (FTMS) in the positive matrix-assisted laser desorption/ionization (MALDI) mode [64]. Samples (0.4 µL) were spotted on a steel plate with 0.4 µl of a 2,5-Dihydroxybenzoic acid matrix (Sigma Aldrich) and air-dried at room temperature and irradiated by a series of 25 impulses at 355 nm from the third harmonic of a neodymium-doped yttrium aluminium garnet (Nd:YAG) laser. The laser power was set to the minimum level necessary to generate a reasonable signal. The signals from the 25 shots were recorded. A ProteoMass Peptide MALDI-MS Calibration Kit (Sigma Aldrich) was used for external calibration. For internal mass calibration, the residual trypsin peak (842.50940 Da) was used. Analysis of the mass spectrometry data was carried out using FTDocViewer software (Varian) and proteins were identified using a Mascot peptide mass fingerprint software program (www.matrixscience.com). The initial search parameters allowed a mass error of up to ±5 ppm and a double trypsin-missed cleavage. Besides, the Protein Prospector MS-Fit software was used to identify the proteins and their PTMs (http://prospector.ucsf.edu). The data sets from three MALDI FT-ICR mass spectrometry were combined.

## Figures and Tables

**Table 1:** Potential modifications of cellular and extracellular proteasomes identified by MALDI FT-ICR MS.

Proteasome		Peptide	Start	End
Cellular	Extracellular			
α1 (PSMA6)				
K45-ac		(R)GKDCAVIVTQK(K)	44	54
T52-p		(R)GKDCAVIVTQK(K)	44	54
		(R)GKDCAVIVTQKK(V)	44	55
*K54-ac*		(R)GKDCAVIVTQK(K)	44	54
T231-p		(R)ILTEAEIDAHLVALAERD(-)	229	246
α2 (PSMA2)				
T48-p		(K)AANGVVLATEKK(Q)	40	51
K53-ac		(K)QKSILYDER(S)	52	60
K70-ub		(K)VEPITKHIGLVYSGMGPDYR(V)	65	84
K227-ub		(R)RLTPTEVKDYLAAIA(-)	220	234
***K227-ac***		(R)LTPTEVKDYLAAIA(-)	221	234
α3 (PSMA4)				
S188-p		(K)SALALAIK(V)	188	195
*K195-ac*		(K)SALALAIK(V)	188	195
K205-ac *		(K)TMDVSKLSAEK(V)	200	210
K210-ac		(K)TMDVSKLSAEK(V)	200	210
		(K)LSAEK(V)	206	210
S207-p		(K)LSAEK(V)	206	210
*K222-ac*		(K)VEIATLTRENGK(T)	211	222
		(K)VEIATLTRENGKTVIR(V)	211	226
K222-ub		(K)VEIATLTRENGKTVIR(V)	211	226
*K222-sc*		(R)ENGKTVIR(V)	219	226
α4 (PSMA7)				
S49-p		(K)KSVAKLQDER(T)	48	57
K52-ub		(K)SVAKLQDER(T)	49	57
*K52-sc*		(K)SVAKLQDERTVR(K)	49	60
S167-p		(K)ANAIGRGAKSVREFLEK(N)	158	174
	S201-p	(K)ALLEVVQSGGK(N)	194	204
	Y228-p	(K)YVAEIEKEKEENEK(K)	228	241
α5 (PSMA5)				
	S79-p	(K)IVEIDAHIGCAMSGLIADAK(T)	67	86
S197-p		(K)EAIKSSLIILKQVMEEK(L)	193	209
		(K)SSLIILKQVMEEK(L)	197	209
S198-p *		(K)EAIKSSLIILKQVMEEK(L)	193	209
		(K)SSLIILKQVMEEK(L)	197	209
	K239-ac *	(K)EELEEVIKDI(-)	232	241
α6 (PSMA1)				
	S40-p	(K)SKTHAVLVALKR(A)	40	51
	*K41-ac*	(K)SKTHAVLVALKR(A)	40	51
	*K41-sc*	(K)SKTHAVLVALKR(A)	40	51
	T42-p	(K)SKTHAVLVALKR(A)	40	51
	*K50-ac*	(K)SKTHAVLVALKR(A)	40	51
	*K50-sc*	(K)SKTHAVLVALKR(A)	40	51
S54-p		(K)RAQSELAAHQKK(I)	51	62
	K62-sc	(K)KILHVDNHIGISIAGLTADAR(L)	62	82
α7 (PSMA3)				
*K29-ac*		(R)VFQVEYAMK(A)	21	29
*K43-ac*		(K)AVENSSTAIGIRCK(D)	30	43
S56-p		(R)CKDGVVFGVEKLVLSK(L)	42	57
		(K)LVLSK(L)	53	57
K57-ac		(K)LVLSK(L)	53	57
K65-ac		(K)LYEEGSNKR(L)	58	66
S87-p		(R)SLADIAR(E)	87	93
T175-p		(R)QAAKTEIEKLQMK(E)	171	183
T186-p		(R)QAAKTEIEKLQMK(E)	171	183
		(K)EMTCRDIVKEVAK(I)	184	196
K192-ac *		(K)EMTCRDIVK(E)	184	192
	K208-sc	(K)DKAFELELSWVGELTNGR(H)	207	224
K230-ac		(R)HEIVPK(D)	225	230
β2 (PSMB7)				
K31-ub		(R)NAVLEADFAKR(G)	22	32
Y34-p		(K)RGYKLPK(V)	32	38
		(R)GYKLPKVR(K)	33	40
K35-ac		(R)GYKLPKVR(K)	33	40
K38-ac		(R)GYKLPKVR(K)	33	40
	T61-p	(K)DGIVLGADTRATEGMVVADK(N)	53	72
	T64-p	(K)DGIVLGADTRATEGMVVADK(N)	53	72
*K72-ac*		(K)DGIVLGADTRATEGMVVADK(N)	53	72
T121-p *		(R)VVTANRMLK(Q)	119	127
	*K225-ac*	(K)NKLDFLRPYTVPNKK(G)	224	238
	Y232-p	(K)NKLDFLRPYTVPNKK(G)	224	238
	T233-p *	(K)NKLDFLRPYTVPNKK(G)	224	238
	K237-ac *	(K)NKLDFLRPYTVPNKK(G)	224	238
		(K)LDFLRPYTVPNK(K)	226	237
		(K)LDFLRPYTVPNKKGTR(L)	226	241
	**K238-ac**	(K)NKLDFLRPYTVPNKK(G)	224	238
		(K)LDFLRPYTVPNKKGTR(L)	226	241
T240-p		(K)GTRLGR(Y)	239	244
β3 (PSMB3)				
*K41-ac*		(R)RFGIQAQMVTTDFQK(I)	27	41
K115-ac		(K)RFGPYYTEPVIAGLDPK(T)	99	115
	***K192-ac***	(R)DAVSGMGVIVHIIEK(D)	178	192
	S181-p *	(R)DAVSGMGVIVHIIEKDK(I)	178	194
**K201-ub**		(R)TLKARMD(-)	199	205
β4 (PSMB2)				
	S23-p	(R)VAASNIVQMKDDHDKMFK(M)	20	37
	K29-ac *	(R)VAASNIVQMKDDHDKMFK(M)	20	37
*K34-ac*		(R)VAASNIVQMKDDHDKMFK(M)	20	37
		(K)DDHDKMFK(M)	30	37
*K37-ac*		(R)VAASNIVQMKDDHDKMFK(M)	20	37
		(K)DDHDKMFK(M)	30	37
	K198-ub	(K)NGIHDLDNISFPKQGS(-)	186	201
β5 (PSMB5)				
	K130-ub	(R)IYELRNKER(I)	124	132
K130-ac		(R)NKERISVAAASK(L)	129	140
S134-p		(R)NKERISVAAASK(L)	129	140
S139-p		(R)NKERISVAAASK(L)	129	140
*K140-ac*		(R)NKERISVAAASK(L)	129	140
β6 (PSMB1)				
K70-ac		(R)LSEGFSIHTRDSPK(C)	57	70
	Y103-p	(K)IIEARLKMYK(H)	95	104
S209-p		(R)AMRLVKDVFISAAER(D)	199	213
		(R)LVKDVFISAAER(D)	202	213
K204-ac		(R)LVKDVFISAAER(D)	202	213
*K228-sc*		(R)DVYTGDALRICIVTKEGIR(E)	214	232
*K228-ac*		(R)ICIVTKEGIREETVSLR(K)	223	239

The sites of PTM identified for the first time are underlined. The PTM sites in italics indicate that other PTMs have previously been found at these amino acid residues. p—phosphorylation; ac—acetylation; ub—ubiquitination; sc—succinylation, *—these PTMs were identified only in mouse or rat proteasome.

## Supplementary Materials

The following are available online at https://www.mdpi.com/1420-3049/25/15/3504/s1, Table S1: The data of MALDI FT-ICR MS analysis of cellular and extracellular proteasomes, Table S2: The results of MALDI FT-ICR MS analysis of cellular and extracellular proteasomes, Table S3: Potential modifications of cellular and extracellular proteasomes identified by MALDI FT-ICR MS.

## Abbreviations

FT-ICR MS—Fourier transform ion cyclotron resonance mass spectrometry; MALDI—matrix-assisted laser desorption/ionization; PTM—post-translational modification; ac—Acetylation; ub—Ubiquitination; p—Phosphorylation; sc—Succinylation; TEV—tobacco etch virus.
